# MicroRNA 21a-5p overexpression impacts mediators of extracellular matrix formation in uterine leiomyoma

**DOI:** 10.1186/s12958-018-0364-8

**Published:** 2018-05-11

**Authors:** Eden R. Cardozo, Rosemary Foster, Anatte E. Karmon, Amy E. Lee, Leah W. Gatune, Bo R. Rueda, Aaron K. Styer

**Affiliations:** 1 Vincent Center for Reproductive Biology, Vincent Department of Obstetrics and Gynecology, Massachusetts General Hospital, 55 Fruit Street, Yaw 10A, Boston, MA 02114 USA; 2 Department of Obstetrics, Gynecology, and Reproductive Biology, Harvard Medical School, Boston, MA 02115 USA; 3 Women and Infants Fertility Center, Brown University Warren Alpert Medical School, 90 Plain Street, Providence, RI 02905 USA

**Keywords:** Fibroids, Leiomyoma, miR-21, Extracellular matrix, TGF-β

## Abstract

**Background:**

MicroRNAs (MiR) may promote fibroid development via altered expression of genes involved in cell proliferation and ECM formation, and evidence supports aberrant expression of MicroRNA (MiR) 21a-5p in fibroids. The purpose of this study was to investigate the functional significance of MiR 21a-5p overexpression in the pathobiology of leiomyomata (fibroids).

**Methods:**

A basic science experimental design using immortalized fibroid and myometrial cell lines derived from patient-matched specimens was used. Stable overexpression of MiR-21a-5p in an immortalized fibroid and patient matched myometrial cell line was achieved through lentiviral vector infection. Main outcome measures were MiR-21-5p overexpression, target gene and protein expression, collagen (COL1A1) production, cell proliferation, cell migration, and cell cycle stages of fibroid and myometrial immortalized cell lines.

**Results:**

MiR-21a-5p was overexpressed to similar levels in fibroid and myometrial cell lines after lentiviral infection. Increased expression of miR-21 resulted in increased gene and protein expression of TGF-β3 in both fibroid and myometrial cells. Changes in expression of the ECM genes Fibronectin, Collagen 1A1, CTGF, Versican and DPT were seen in both fibroid and myometrial cells. Changes were also seen in Matrix Metalloproteinase (MMP) related genes including MMP 2, MMP 9, MMP 11 and Serpine 1 in both fibroid and myometrial cells. MiR-21 upregulation resulted in increased proliferation and migration in fibroid cells compared to myometrial cells.

**Conclusions:**

MiR-21a-5p overexpression results in changes in the expression of ECM mediators in both fibroid and myometrial cells, and increased cell proliferation in fibroid cells. These finding suggest a potential functional role of MiR-21a-5p in the development of uterine fibroids and warrant further investigation.

**Electronic supplementary material:**

The online version of this article (10.1186/s12958-018-0364-8) contains supplementary material, which is available to authorized users.

## Background

Uterine fibroids, or leiomyoma, represent a major public health problem for women of reproductive age. These benign smooth muscle tumors of the myometrium have a cumulative incidence of nearly 70% in white women and over 80% in black women [1]. In the United States, fibroids remain the leading indication for hysterectomy [2], and are estimated to confer a total annual societal cost of up to $34.4 billion [3]. Although this is one of the most commonly treated gynecologic disorders, there are no consistently effective medical treatments to prevent fibroid development or to reduce the risk of recurrence. Limitations in current medical treatment underscore the need to improve our understanding of the pathobiology of leiomyoma and to investigate disease-relevant therapeutic targets.

The most commonly accepted etiology for uterine fibroid development is the transformation of myometrial smooth muscle fibroblasts and dysfunctional extracellular matrix (ECM) formation [4]. Transforming growth factor beta (TGF-β) and its cognate receptor (TGF-βR), have been implicated by many investigators as key mediators of aberrant cell signaling which contributes to the development of disease [4, 5]. Emerging evidence indicates that many additional genetic and epigenetic alterations may regulate the formation of leiomyomas [6, 7]. Specifically, microRNAs (miRs) have been recently implicated as epigenetic mediators in the pathogenesis of fibroids [8]. It has been suggested that miRs may promote fibroid development via altered expression of genes responsible for proliferation, apoptosis, angiogenesis, and ECM formation [7].

MiRs are small, non-coding, stable RNAs approximately 22 base pairs long [9], and are thought to regulate gene expression via gene silencing with either inhibition of translation or degradation of target messenger mRNA [8]. Studies have also demonstrated that a minority of miRs may activate gene targets by directly binding to their promoter regions and result in upregulation of target genes [10]. There is substantial evidence that aberrant miR expression plays a role in the development of multiple malignancies and benign human diseases [8, 11–13]. Differential expression of several miR species in fibroids compared to matched native myometrium has implicated these small non-coding RNAs as possible mediators in the pathobiology of fibroids [14–17].

Overexpression of miR-21 in fibroids (compared to myometrium) has been observed by several independent investigators [8, 16, 18]. Elevated miR-21 has also been reported in several models in cancer biology, and this miR has been shown to inhibit tumor suppressors, to increase cell proliferation, and to promote tumorigenesis [19–22]. Overexpression of miR-21 has also been observed in myocardial, renal, and pulmonary fibrosis models [23–26]. It is considered a “profibrogenic miR” since it has been shown to target the TGF-β downstream signaling inhibitor Smad-7 and to accentuate the profibrotic function of TGF signaling mediators in promoting excessive ECM formation in hepatic cells [27].

Recent evidence suggests that various miRs may regulate cell proliferation and ECM formation in uterine fibroids [15, 28]. Given the known role of miR-21 in targeting tumor suppressors, regulation of the profibrotic TGFβ pathway in ECM production, and consistent reports of aberrant expression of miR-21 in fibroids in several studies, further investigation of this miR has the potential to delineate the role of miRs in the development of fibroids. The objective of this study is to determine the functional significance of miR-21 overexpression in the pathobiology of uterine leiomyoma. We hypothesize that miR-21 overexpression in uterine fibroid and myometrial cells will impact the mediators of the TGFβ pathway, and alter the expression of regulators of ECM production.

## Methods

This study was approved by the Partners Healthcare Human Research Committee /Institutional Review Board.

### Cell lines / cell culture

An immortalized fibroid cell line (A006-X) and patient-matched myometrial cell line (A005-X) was obtained from the Henry M. Jackson Foundation for the Advancement of Military Medicine, Inc. as previously described [29–31]. Cells were maintained at 37 °C in DMEM-F12 supplemented with 10% fetal bovine serum (FBS), Amphotericin-B anti-fungal reagent, Penicillin/Streptomycin antibiotic, and GlutaMAX supplement (Thermo Fisher Scientific, Waltham, MA).

### Lentiviral infections

Stable miR-21a-5p overexpression of the fibroid cell line was achieved using a SMART choice shMIMIC ™ lentiviral microRNA vector (GE Dharmacon, Lafayette, CO) with GFP reporter, non-targeting (NTC) negative controls and Glyceraldehyde-3-Phosphate Dehydrogenase (GAPDH) positive controls according to manufacturer’s protocols. Briefly, cells were plated in triplicate in 96 well plates and dilutions of lentiviral particles were prepared to achieve multiplicity of infection (MOI) of 1.0, 2.5 and 5.0 respectively. Cells were incubated overnight with transduction medium (1:1 mixture of DMEM with cell culture medium as described above, plus polybrene) containing lentiviral particles then the following day cell culture medium was added to wells and plates were returned to the incubator for 96 h (hr). Knockdown of GAPDH positive control at the mRNA level was verified with RT-qPCR, and fluorescent microscopy was used to confirm efficient infection via TurboGFP™ expression. Establishment of a stable cell line overexpressing miR-21a-5p as well as a stable cell line of NTC was accomplished by selecting for puromycin-resistant cells. RT-qPCR (has-miR-21-5p LNA PCR primer set; Exiqon, Vedbaek, Denmark) was used to confirm over-expression of miR-21a-5p in the fibroid and myometrial cell lines. Infections were performed in biological triplicate. For all subsequent experiments, fibroid cells infected at MOI of 5 and myometrial cells infected at MOI of 2.5 were used, as these resulted in similar upregulation of miR-21a-5p in the respective cell lines.

### RNA isolation

RNA was isolated from fibroid and myometrial cells using the mirVana isolation kit (Life Technologies, Carlsbad, CA) according to manufacturer instructions. RNA concentration was quantified using the Nanodrop 2000 Spectrophotometer (ThermoFisher Scientific, Waltham, MA).

### MiR expression real-time qPCR

MiR polyadenylation and reverse transcription to cDNA was performed using the miRCURY LNA microRNA Universal cDNA synthesis kit (Exiqon) per manufacturer protocol. RT-qPCR was performed according to manufacturer protocol using miRCURY LNA microRNA PCR ExiLENT SYBR Green master mix (Exiqon) and has-miR-21-5p LNA PCR primer set. In order to detect RNA contamination, a non-targeting negative control (NTC) was included in the RT step. Artificial RNA (Sp6) was added to the reverse transcription step as a positive control to confirm that reverse transcription and amplification occurred equally in all samples. MiR-30c, a ubiquitously expressed miR, was used as an internal control. CFX96 Real-Time PCR Detection System (BioRad, Hercules, CA) was used for DNA amplification. Fluorescence of SYBR green dye bound to dual-stranded DNA was utilized for detection. The real-time thermal cycle program was 95°C for 30 s (s) followed by 40 cycles of 95°C for 5 s and 58°C for 30 s.

Experiments were repeated in technical triplicates for each of three infections. Relative changes in MiR expression were calculated using the standard 2^-ΔΔ**CT**^ method [32].

### Gene target expression real-time qPCR

Isolated RNA was used to synthesize cDNA using the Superscript VILO cDNA kit (ThermoFisher Scientific, Cambridge, MA). CFX96 Real-Time PCR Detection System (BioRad, Hercules, CA) was used for DNA amplification. Fluorescence of SYBR green dye bound to dual-stranded DNA was utilized for detection. The real-time thermal cycle program was 95°C for 30 s followed by 40 cycles of 95°C for 5 s and 58°C for 30 s. Probes, purchased from Invitrogen (Waltham, MA), were designed in house. Resultant levels were compared to those determined in control cells infected with a lentivirus encoding a missense miR (non-targeting control, or NTC). Beta-actin was used as the internal control. Experiments were repeated in technical triplicates for each of three infections. Relative changes in gene expression were calculated using the standard 2(−Delta Delta C(T)) method [32].

### Western immunoblot

Immunoblot analysis was performed using protocol as previously described [33]. Antibodies directed against TGFβ3 (Santa Cruz Biotchnology, Dallas, TX) and GAPDH (Cell Signaling Technology, Danvers, MA) were used. The dilution for TGFβ3 was *1:1000* and for GAPDH *1:10,000*. A chemiluminescent detection reagent (ECL Prime, GE Healthcare Life Sciences, Pittsburgh, PA) was used to develop the blots and then images were created using the Bio Rad ChemiDoc XRS + Imaging System (BioRad, Hercules, CA) and subsequently analyzed using ImageJ software (National Institutes of Health, Bethesda, MD). The level of TGF-β3 was quantitated to the level of GAPDH (loading control).

### Collagen (COL1A1) assay

The amount of type I collagen produced by fibroid and myometrial cells over-expressing miR-21a-5p, as well as non-targeting controls, was quantified using the Human Type I Collagen Detection Kit (Chondrex, Inc., Redmond, WA) with ELISA according to the manufacturers instructions. Cells were pretreated with pepsin and elastase and then capture antibody solution was added to cells and they were incubated overnight at 4 °C. Serial dilutions of standard solution were prepared, samples and standard tubes were mixed, incubated at room temperature for 2 h, then plates were washed and detection antibody was added. Steptavidin Peroxidase was added to each well, and cells were incubated for 1 h, after which the plate was washed and OPD solution (o-phenylenediamine dihydrochloride in chromagen dilution buffer) was added. Cells were incubated for 30 min at room temperature then 50 μl of 2 N sulfuric acid was added to each well. The OD values were read at 490 nm. The concentration of collagen (μg/ml) was plotted against standard values provided by the manufacturer, and the amount human type I collagen in samples was calculated using regression analysis. Experiments were repeated in technical triplicate for each infection.

### Cell proliferation assay

Proliferation was determined by plating the cells in 24-well plates and culturing as described above. Fibroid and myometrial cells over-expressing miR-21-5p and non-targeting controls were utilized. Fibroid cells were plated at 5000 cells/well and myometrial cells were plated at 10,000 cells/well. Cells were incubated for 24 and 48 h, cells harvested by trypsinization, and the number of viable cells per milliliter was determined by trypan blue exclusion (Invitrogen, Carlsbad, CA) and cell counting using a Bio-RAD TC10™ Automated Cell Counter. Live cells were counted at 24 and 48 h respectively.

### Cell cycle analysis

Fibroid and myometrial cells over-expressing miR-21a-5p and non-targeting controls were cultured and then cells were arrested in mitosis by blocking with thymidine, releasing, and then blocking with nocodazole. To accomplish this, when cells reached 40% confluence, 2 mM thymidine was added and then cultured for 24 h. Thymidine was then removed by washing with 1xPBS and adding fresh DMEM (culture media) for 3 h in order to release the cells. Nocodazole (100 μg/ml) was then added to the media for 12 h then removed by washing with 1xPBS and fresh culture media was added. Cells were harvested and fixed in cold 70% ethanol, then centrifuged and washed twice with PBS. Cells were stained with Live/Dead Fixable Far Red Dead Cell Stain Kit (ThermoFisher Scientific, Waltham, MA) to eliminate dead cells during LSR analysis. Ribonuclease A was added directly to the pellet, then DAPI (1 μg/ml) was added directly to cells in ribonuclease A solution and incubated for 15 min at room temperature. Cells were examined on a BD LSRII analyzer, and cell cycle analysis was performed using FlowJo software (Treestar Inc., Ashland, OR). Experiments were repeated in technical triplicate for each infection.

### Migration assay

Fibroid and myometrial cells over-expressing miR-21a-5p and non-targeting controls were utilized in the transwell cell migration assay model. Fibroid cells were plated at 5000 cells/well and myometrial cells were plated at 10,000 cells/well due to faster rate of growth of fibroid cells. Cells that migrated across the membrane (sterile 6.5 mm Corning Transwell with 8um pore polycarbonate membrane inserts [Coring Inc., Coring, NY]) were fixed with Crystal Violet staining solution (Sigma-Aldrich, Natick, MA) and counted at 2.5 h.

### Statistical analysis

For qPCR analyses, comparisons between tissue types were made utilizing ddCP values (fold change, with either myometrium or NTC as reference group). Statistical significance was evaluated with unpaired t-tests and 2-way ANOVA. Western immunoblot results are presented as relative to the NTC and statistical significance was evaluated using an unpaired t-test. Collagen ELISA results are presented as absolute numbers and results are analyzed with a 2-way ANOVA. Cell cycle and proliferation assay results for the miR21 infected cell line derivatives are presented relative to the results obtained for the corresponding NTC and also analyzed with 2-way ANOVA. Migration data are similarly presented as relative numbers and analyzed using an unpaired t-test. Data were analyzed using Prism software (GraphPad Software Inc., La Jolla, CA).

## Results

### MiR-21 expression

**Fig. 1:**
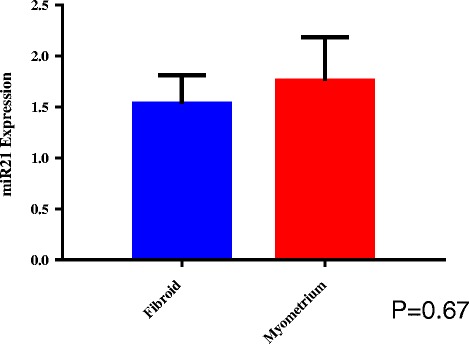
Relative Expression of miR-21 in Fibroid and Myometrium. RT-PCR relative expression of miR-21 in fibroid and myometrium after infection with lentiviral vector. Results are presented as fold change with non-targeting controls (NTC) as the reference, mean ± SEM of three independent experiments from each of three independent infections. *P*-value < 0.05 is indicated by an asterisk

Figure 1 shows the relative expression of miR-21 in fibroid and myometrium after infection with lentivirus based on real time PCR. Results are normalized to a non-targeting control (NTC). The level of miR-21 upregulation was similar between fibroid and myometrium following lentiviral infection.

### TGF-β family gene and protein expression

**Fig. 2:**
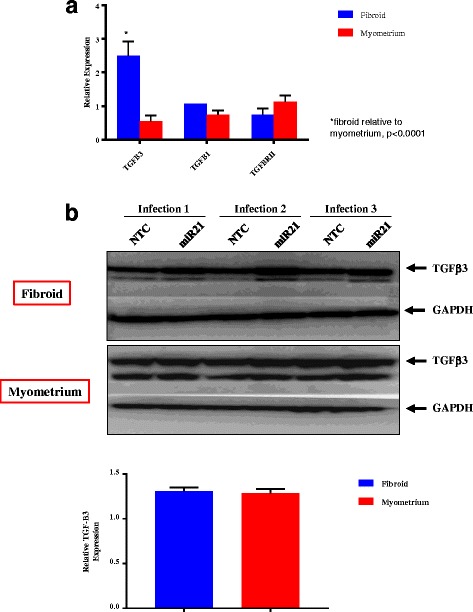
Gene and protein expression of TGF-β family. All results are presented as fold change with NTC as the reference, mean ± SEM, and *P*-value < 0.05 is indicated by an asterisk. **a** Expression of TGF-β Family Genes. Gene target RT-PCR relative expression of TGF-β family genes in fibroid and myometrial cells upregulated with miR-21. Results represent the mean of three independent experiments from each of three independent infections. **b** TGF-β3 protein expression. Western Blot protein expression of TGF-β3 in fibroid and myometrial cells upregulated with miR-21. GAPDH is shown as loading control. Graph shows TGF-β3 protein expression (corrected for GAPDH levels) in miR-21 upregulated fibroid and myometrium is normalized to each tissue’s respective NTC (corrected for GAPDH levels). Results represent the mean of three independent experiments

Relative levels of gene expression for TGF-β3, TGF-β1 and TGF-βRII respectively in fibroid and myometrium (compared to their respective NTCs) are shown in Fig. 2a. TGF-β3 gene expression was greater in miR-21 upregulated fibroid cells compared to miR-21 upregulated myometrial cells (*p* < 0.0001). An increase was observed in TGF-β3 gene expression in miR-21upregulated fibroid cells relative to their NTC (*p* = 0.04). Similar levels of TGF-β1 and TGF-βRII expression were observed in the infection and NTC group in both fibroids and myometrium respectively.

Assessment of TGF-β3 protein expression via Western blot in miR-21 upregulated fibroid and myometrial cells is presented in Fig. 2b. TGF-β3 protein expression was higher in miR-21 upregulated fibroid compared to NTC (*p* = 0.0149) and in miR-21 upregulated myometrium compared to NTC (*p* = 0.0002) (Additional file 1: Figure S1). When TGF-β3 protein expression (corrected for GAPDH levels) in miR-21 upregulated fibroid and myometrium is normalized to each tissue’s respective NTC (corrected for GAPDH levels), there is no significant difference in TGF-β3 protein expression between fibroid and myometrium (*P* = 0.73) (Fig. 2b).

### Gene expression of matrix metalloproteinase (MMP) genes

**Fig. 3:**
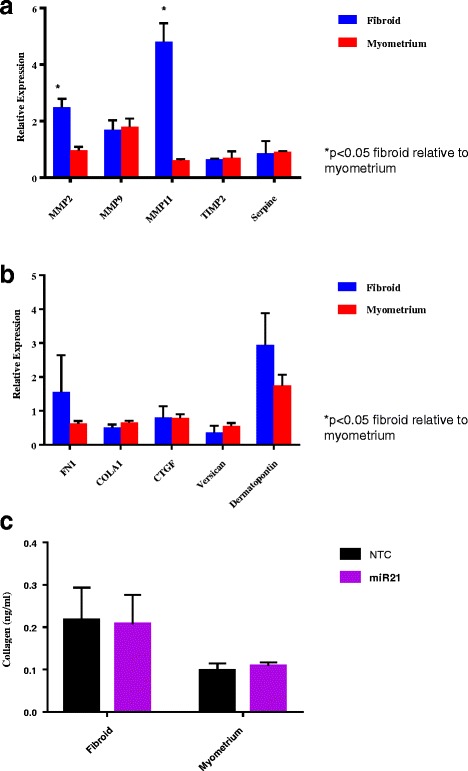
Gene and protein expression of Matrix Metalloproteinase (MMP) and Extracellular Matrix (ECM) family. Results are presented as fold change with NTC as the reference, mean ± SEM of three independent experiments from each of three independent infections. P-value < 0.05 is indicated by an asterisk. **a** MMP family. Gene target RT-PCR relative expression of MMP family genes in fibroid and myometrial cells upregulated with miR-21. **b** ECM Family. Gene target RT-PCR relative expression of ECM family genes in fibroid and myometrial cells upregulated with miR-21. **c** Collagen Assay. ELISA results of Collagen 1A1 protein expression in fibroid and myometrial cells upregulated with miR-21

Figure 3a shows the impact of miR-21 upregulation on the relative expression of ECM related genes. An increase in MMP 2, MMP 9, MMP 11 and Serpine 1 expression in miR-21 upregulated fibroid cells was observed compared to NTC (*p* = 0.01, 0.009, 0.007 and 0.02, respectively). Decreased expression of MMP-11 (*p* = 0.007) and Serpine 1 (*p* = 0.03) was observed in miR-21 upregulated myometrium compared to NTC. When miR-21 upregulated fibroid cells were compared to miR-21 upregulated myometrium cells, a difference in MMP-2 (*p* = 0.01) and MMP-11 (*p* = < 0.0001) expression respectively was observed.

### Gene expression of extra cellular matrix related genes

The relative gene expression of mediators of ECM formation are presented in Fig. 3b. In miR-21 upregulated fibroid cells, there was a decrease in expression of Collagen 1A1 (*p* = 0.02) and Versican (*p* = 0.02) respectively compared to NTC. In miR-21 upregulated myometrial cells, there was a decrease in the expression of Fibronectin (*p* = 0.02), Collagen 1A1 (*p* = 0.02), CTGF (*p* = 0.01), Versican (*p* = 0.02) and DPT (*p* = 0.04) respectively in the myometrium. No differences were observed when fibroid and myometrium were compared.

### Collagen assay

No difference was seen in collagen accumulation when comparing miR-21 upregulated fibroid or myometrium to NTC or when comparing miR-21 upregulated fibroid with miR-21 upregulated myometrium (Fig. 3c).

### Cell proliferation

**Fig. 4:**
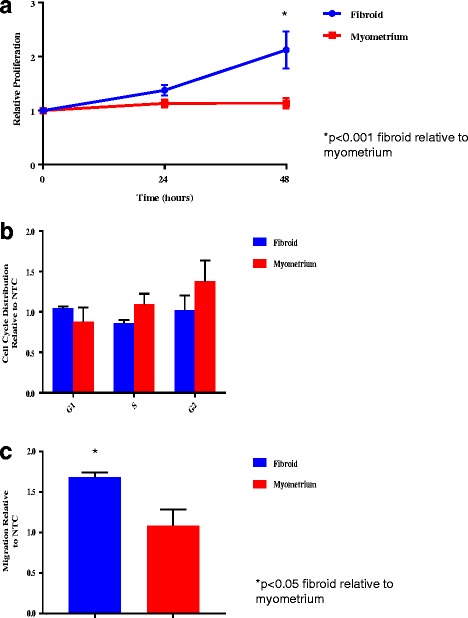
Cell proliferation, cell cycle analysis and relative migration. Results are presented with fibroid and myometrial cells normalized to NTC as the reference. *P*-value < 0.05 is indicated by an asterisk. **a** Proliferation Assay. Cell proliferation at 24 and 48 h time points after plating in fibroid compared to myometrial cells upregulated with miR-21. Average cell proliferation by time point is presented as mean ± SEM of three independent experiments from each of three independent infections. **b** Cell Cycle Analysis. Cell cycle analysis of fibroid compared to myometrial cells upregulated with miR-21. Transition through the cell cycle (G1, S, G2) is presented as mean ± SEM of three independent experiments. **c** Migration Assay. Relative migration of miR-21 upregulated fibroid compared to myometrial cells. Relative migration by time point is presented as mean ± SEM of each of three independent infections of both fibroid and myometrium

In fibroid cells, an increase in cell proliferation in cells with miR-21 upregulation compared to controls (*p* < 0.0001) was observed at the 48 h time point. No differences were observed in myometrial cells at any time point (Additional file 2: Figure S2). Figure 4a depicts the results of cell proliferation in miR-21 upregulated fibroid and myometrial cells (normalized to respective NTCs), measured at 24 and 48 h respectively after plating. There was increased proliferation in fibroid cells as compared to myometrial cells at 48 h after plating (*p* < 0.001).

### Cell cycle analysis

Cell cycle analysis of miR-21 upregulated fibroid compared to myometrial cells (normalized to respective NTCs) are shown in Fig. 4b. Cell cycle analysis of miR-21 upregulated fibroid and myometrial cells compared to their respective NTCs are shown in Additional file 3: Figure S3. Upregulation of miR-21 did not have any statistically significant impact on transition through the cell cycle, in fibroid compared to myometrium and fibroid or myometrium compared to their respective NTC.

### Migration assay

Migration of miR-21 upregulated fibroid compared to myometrial cells (normalized to respective NTCs) is presented in Fig. 4c. Increased migration in miR-21 upregulated fibroid cells compared to myometrial cells (*p* = 0.0467) was observed. No difference was observed in the percentage of miR-21 upregulated fibroid cells migrated relative to the fibroid NTC cells, nor in the percentage of myometrial cells migrated compared to their matched myometrial NTCs (Additional file 4: Figure S4).

## Discussion

This study examined the impact of miR-21 overexpression on regulators of ECM formation and functional endpoints in fibroid and myometrial cells. Increased gene and protein expression of TGF-β3, and altered gene expression of several well-described mediators of the ECM in both fibroid and myometrial cells was observed. In addition, several cell specific phenotypic changes, including increased proliferation and migration in fibroid cells compared to myometrial cells myometrial cells occurred following upregulation of miR-21.

Given the phenotypic signature of fibroids, aberrant ECM production and progressive growth, our findings expand the understanding of the possible functional significance of miR-21 in the pathobiology and genesis of this reproductive disorder. Although miR-21 overexpression in fibroids has been reported by other investigators [16, 18], several studies that evaluated the role of miR-21-5p in the pathogenesis of leiomyoma have been previously retracted [34, 35], and the functional significance of this miR in fibroid biology has been incompletely studied [36, 37]. To our knowledge, our study is the first to provide evidence that miR-21 impacts ECM mediators such as TGF-β3 and MMPs in uterine fibroids.

TGF-β3 has been established as a significant regulator of ECM formation in uterine fibroids [4, 5]. TGF-β is a growth factor that promotes connective tissue formation, and the TGF-β3 has been shown to have 3 to 4-fold higher expression in fibroids compared to matched myometrium [38, 39]. The intrinsic biological function of TGF-β, which includes cellular hypertrophy and ECM turnover, are central to various fibrotic disorders, and may be essential to fibroid development and progression [4]. Notably, the use of TGF-β signaling inhibitors in rodent fibroid models results in decreased incidence and multiplicity of uterine leiomyoma [40]. Based upon the findings of this study, overexpression of miR-21 in fibroids may play a role in TGF-β3 mediated ECM formation in fibroids.

The increased expression of TGF-β3,the primary isoform of TGF-β found in the uterus [41], and in leiomyoma cells, appears to have an effect on genes encoding collagen formation [38] and several other genes which influence ECM formation. To this end, it may play a role in the excessive and dysregulated ECM production observed in fibroids. Studies exploring the impact of culturing immortalized fibroid and myometrial cells with TGF-β3 found increased mRNA and protein production of ECM proteins collagen 1A1 [29, 42], fibronectin 1 [29, 42] and connective tissue growth factor [29] in both cell types, with increased expression in treated myometrial cells to nearly the levels found in leiomyoma cells [29]. Interestingly, in our study, the effect of increased miR-21 and the subsequent increased expression of TGF-β3 in fibroid cells had the opposite impact, and resulted in decreased expression of COL 1A1 and versican. In myometrial cells, increased miR-21 expression resulted in decreased expression of fibronectin, collagen 1A1, CTGF, versican and DPT. A possible explanation for this difference in results between our study and others is that miR-21 may directly and indirectly target several upstream and downstream mediators of the TGF-β pathway, and may result in variable expression of ECM related genes. As a result, further investigation is warranted to evaluate if increased TGF-β3 expression is the result of direct targeting of miR-21 or an indirect on mediators of TGF-β signaling.

Treatment of fibroid and myometrial cells with TGF-β3 has also been shown to result in increased expression of GAG-rich versican variant, which plays an important structural role in ECM organization and also influences growth and proliferation [39]. While our study showed a decrease in versican expression, fibroid cells with upregulated miR-21 expression, similar to prior studies, did have a significantly increased rate of proliferation. An increase in proliferation is notable since aberrant proliferation is a hallmark of fibroid cells. Notably, migration was increased in miR-21 upregulated fibroid cells compared to myometrium. Given that there was no difference in the amount of miR-21 upregulation between fibroid and myometrial cells in this study, these differences may suggest that the functional significance of this miR may be specific to cell type.

Treatment of fibroid cells with TGF-β3 has also been shown to induce increased expression of Serpine-1 (plasminogen activator inhibitor-1) protein [42], an inhibitor of matrix-metalloproteinases (MMP). This is consistent with studies showing TGF-β3 results in decreased expression of genes for MMP-2 and MMP-11, involved in matrix resorption [29]. In contrast, miR-21 upregulated fibroid cells in this study have increased expression of MMP 2, MMP 9, MMP 11 and Serpine-1, in spite of the associated increase in TGF-β3 expression. Again this may reflect the impact on miR-21 at additional targets throughout the TGF-β pathway, or at other sites, which impact the expression of ECM-related genes. Furthermore, miR-21 upregulation in myometrial cells resulted in decreased expression of MMP 11 and Serpine 1, providing further evidence that the role of miR-21 differs among specific tissue/cell types.

While this study highlights the potential impact of miR-21 and TGF-β3 expression in uterine fibroids, further investigation is necessary to better delineate the impact of miR-21 on fibroid pathobiology, especially as it relates to the TGF-β3 pathway and the extracellular matrix. Strengths of this study include the use of patient matched fibroid and myometrial cell lines. Long-term lentiviral infection, as opposed to short term transfection, was used to upregulate mir-21 in the fibroid and myometrial cells, which allows for study of the downstream impact of miR-21 over a longer period of time. The functional impact of miR-21 overexpression was examined by evaluating multiple fibroid relevant ECM-related phenotypic and genotypic endpoints, providing a novel preliminary understanding of the role of miR-21 overexpression in the pathogenesis of uterine fibroids.

Limitations of this study include, as previously noted, the fact that a single miR can have multiple simultaneous targets, which can effect multiple signaling pathways. Therefore overexpression of miR-21 may affect expression of a predicted gene target, such as TGF-β3. However, it is unknown if miR-21 is acting directly on this target or via an upstream target in the pathway. Furthermore, as seen with the variable impact of miR-21 on fibroid versus myometrial cells, miRs can act differently in different tissues. Accordingly, the results of this study may not be applicable in other tissue types, and should be interpreted with caution as miR-21 in fibroid and myometrial cells may function differently in vivo versus in vitro. Since this study utilized in vitro models, it is difficult to evaluate the true impact of endogenous sex steroids. Future in vivo studies are needed to understand the influence of endogenous sex steroids on the miR-21 and its role in fibroid pathobiology*.* Future long-term studies should also aim to assess if miR-21 may be a target for therapeutic intervention that may cause regression of the fibroid phenotype to the normal tissue state. If so, studies will need to determine the most effective way of targeting miR-21 in fibroid tissue only, given that miR-21 is fairly ubiquitous in human tissue.

## Conclusions

In summary, this study has shown that that upregulation of miR-21 resulted in increased gene and protein expression of TGF-β3, altered gene expression of several mediators of the ECM in both fibroid and myometrial cells, and phenotypic changes including increased proliferation and migration in fibroid cells. Our findings support the hypothesis that miR-21 may assert its action in fibroid cells in part via the TGF-β3 pathway and adds to the increasing evidence for a role of miR-21 in the pathobiology of fibroids. Since fibroid and myometrial cells do not respond in the same fashion to upregulation of miR-21, it may be inferred that miR-21 that the cellular transition from myometrium to uterine fibroids is not solely regulated by miR-21 overexpression. Given the tremendous morbidity and societal cost of uterine fibroids and dearth of effective medical interventions, identification of novel therapeutic targets is critical. This study highlights the importance of miR-21, perhaps via its role in the TGF-β3 pathway, as a focus of future investigation in fibroid biology and as a potential therapeutic target in the treatment of uterine fibroids.

## Additional files

Additional file 1: **Figure S1.** TGF-β3 protein expression. TGF-β3 protein expression miR-21 upregulated fibroid and upregulated myometrium compared to NTC. Results represent the mean of three independent experiments. (PDF 90 kb)

Additional file 2: **Figure S2.** Proliferation Assay. Cell proliferation at 24 and 48 h time points after plating in fibroid and myometrial cells upregulated with miR-21 compared to their respective NTC. Average cell proliferation by time point is presented as mean ± SEM of three independent experiments from each of three independent infections. (PDF 96 kb)

Additional file 3: **Figure S3.** Cell Cycle Analysis. Cell cycle analysis of miR-21 upregulated fibroid and myometrial cells compared to their respective NTCs. Transition through the cell cycle (G1, S, G2) is presented as mean ± SEM of three independent experiments. (PDF 117 kb)

Additional file 4: **Figure S4.** Migration Assay. Relative migration of miR-21 upregulated fibroid and myometrial cells compared to their respective NTCs. Relative migration by time point is presented as mean ± SEM of each of three independent infections of both fibroid and myometrium. (DOCX 27 kb)

## Abbreviations

ECM: Extracellular matrix
FBS: Fetal bovine serum
GAPDH: Glyceraldehyde-3-Phosphate Dehydrogenase
miRs: microRNAs
MMP: Matrix Metalloproteinase
MOI: Multiplicity of infection
NTC: Non-targeting control
TGF-β: Transforming growth factor beta
TGF-βR: Transforming growth factor beta receptor
