# Supplementary figures and images for: MicroRNA 21a-5p overexpression impacts mediators of extracellular matrix formation in uterine leiomyoma
Source: Reprod Biol Endocrinol. 2018 May 11;16:46. doi: 10.1186/s12958-018-0364-8 (PMC5946472; doi:10.1186/s12958-018-0364-8)

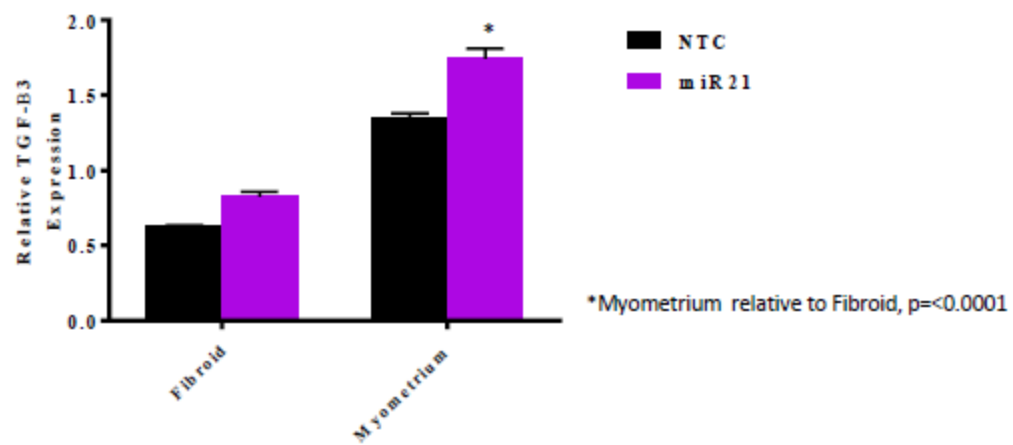

Supplement: Supplementary file 1 — Figure S1. TGF-β3 protein expression. TGF-β3 protein expression miR-21 upregulated fibroid and upregulated myometrium compared to NTC. Results represent the mean of three independent experiments. (PDF 90 kb) [file 12958_2018_364_MOESM1_ESM.pdf]

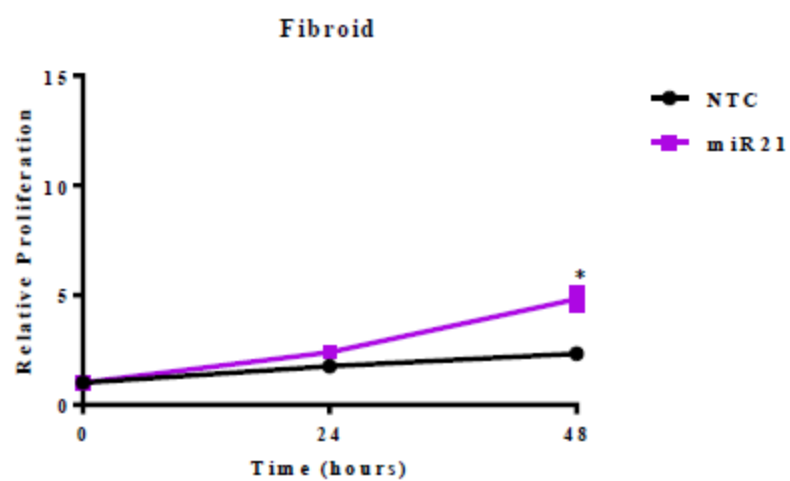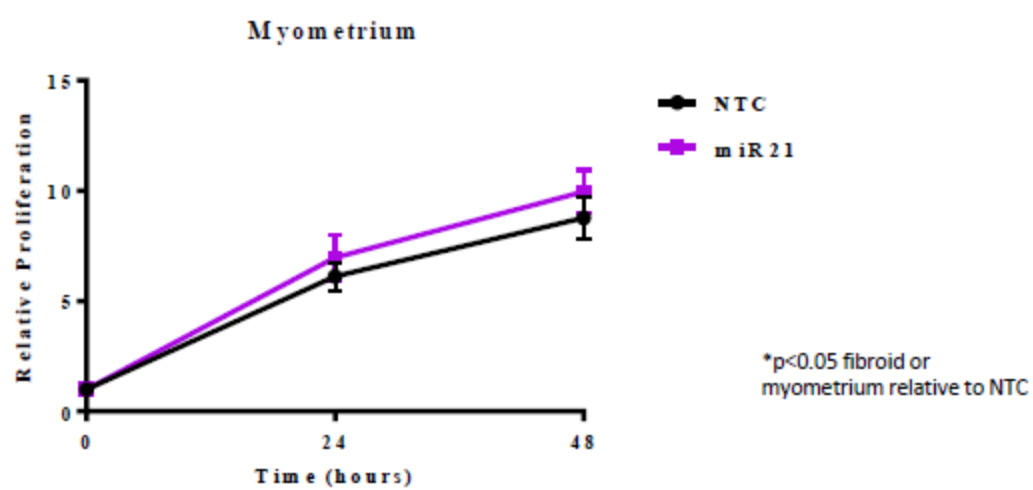

Supplement: Supplementary file 2 — Figure S2. Proliferation Assay. Cell proliferation at 24 and 48 h time points after plating in fibroid and myometrial cells upregulated with miR-21 compared to their respective NTC. Average cell proliferation by time point is presented as mean ± SEM of three independent experiments from each of three independent infections. (PDF 96 kb) [file 12958_2018_364_MOESM2_ESM.pdf]

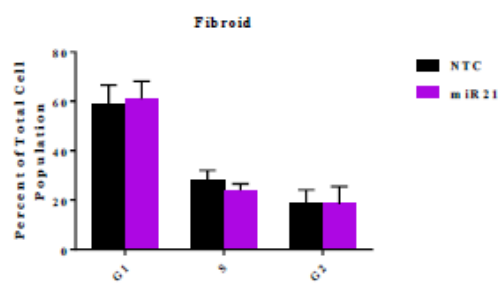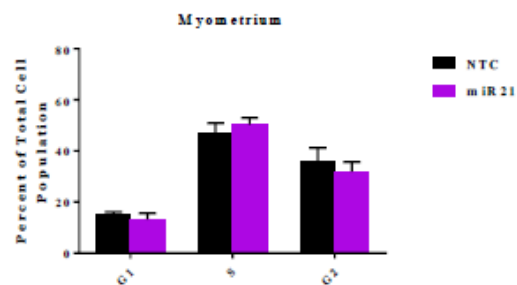

**Control**

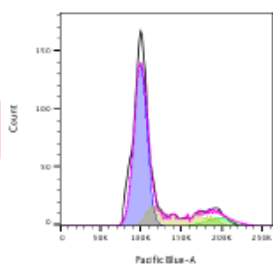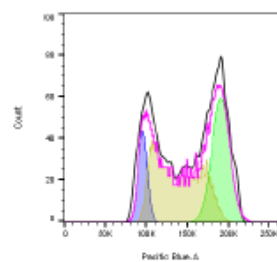

**miR21**

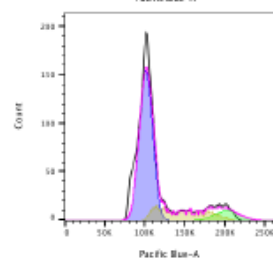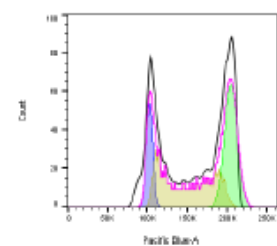

**Fibroid**

**Myometrium**

Supplement: Supplementary file 3 — Figure S3. Cell Cycle Analysis. Cell cycle analysis of miR-21 upregulated fibroid and myometrial cells compared to their respective NTCs. Transition through the cell cycle (G1, S, G2) is presented as mean ± SEM of three independent experiments. (PDF 117 kb) [file 12958_2018_364_MOESM3_ESM.pdf]

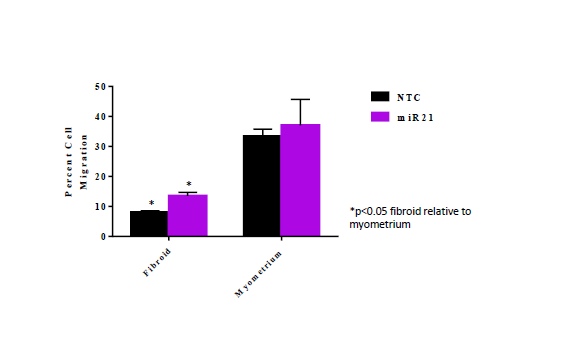

Supplement: Supplementary file 4 — Figure S4. Migration Assay. Relative migration of miR-21 upregulated fibroid and myometrial cells compared to their respective NTCs. Relative migration by time point is presented as mean ± SEM of each of three independent infections of both fibroid and myometrium. (DOCX 27 kb) [file 12958_2018_364_MOESM4_ESM.docx]
